# Destabilizing Interactions in Human Telomeric G‑Quadruplex Multimers

**DOI:** 10.1021/acs.jpclett.5c01100

**Published:** 2025-05-23

**Authors:** Luca Bertini, Mattia Trapella, Deniz Mostarac, Valeria Libera, Caterina Petrillo, Cristiano De Michele, Lucia Comez, Alessandro Paciaroni

**Affiliations:** † Department of Physics and Geology, 9309 University of Perugia, via Alessandro Pascoli, 06123 Perugia, Italy; ‡ Department of Physics, University of Rome La Sapienza, 00185 Rome, Italy; § CNR-IOM c/o Department of Physics and Geology, 27258 University of Perugia, 06123 Perugia, Italy

## Abstract

G-Quadruplexes are secondary structures that may form in G-rich nucleic acid sequences. The extended overhang at the ends of human telomeres has the potential to form multiple G-Quadruplexes that are crucial in regulating key biological processes. Here, we employ small-angle X-ray scattering-guided extremely coarse-grained simulations to provide a picture of the arrangement of G-Quadruplexes in long telomeric sequences. We observe significant destabilizing interactions between G-Quadruplexes, thus making the stacked conformation less prevalent. A helix–coil model is proposed to analytically describe the stacking–unstacking equilibrium within G-Quadruplex multimers and predict the occurrence of stacked and unstacked G-Quadruplex multiplets in arbitrarily long sequences.

## Graphical abstract

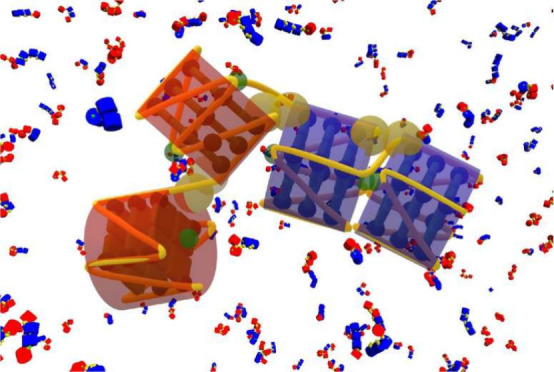

Human telomeres consist of thousands of TTAGGG hexamer repeats along with specialized proteins.^1^ They are located at the termini of eukaryotic chromosomes and are essential for protecting and maintaining the stability of chromosome ends. The terminal region of human telomeres consists of an extended G-rich single-stranded DNA segment of a few hundred bases in nongerm cells, referred to as the 3′-overhang.^2^ Telomeres have been linked to various human diseases, including cancer^3^ and telomeropathies,^4^ as well as to aging^5^ and overall genome stability.^6^ As a concequence, these structures have been identified as potential targets for various therapeutical applications. The single-stranded overhang, which is actively involved in repeated cycles of cell division and the maintenance of genome integrity,^7^ has the potential to form multiple sequentially stacked G-Quadruplex (GQ) units, known as GQ multimers.^8^ GQs consist of stacked G-tetrads, each with guanines at the corners. These guanines form eight Hoogsteen hydrogen bonds per tetrad, and their interactions are stabilized by monovalent cations positioned within or between the tetrad planes.^9^ GQs can adopt three primary topologies —parallel, antiparallel, and hybrid— distinguished by the relative orientation of their four guanine strands and the structural configuration of their loop regions.^10^ The formation of GQ multimers at the human telomeric overhang is thought to serve as a protective capping structure for telomere ends.^11^ The interplay of GQs in the telomeric overhang has been described in terms of the beads-on-a-string arrangement, where the noninteracting units are connected by flexible TTA loops.^12^ An antithetic view is that of GQs forming a macrostructure where each unit interacts with adjacent GQs via stacking interfaces of TTA loops.^13^ Alternative views have been also proposed, where GQs are partially stacked^14^ or repel each other via destabilizing interactions.^15^ As the inter-GQ junction may serve as a binding pocket for ligands, the clarification of the structural properties of GQ multimers is crucial. Certain ligands, such as porphyrins, berberine and actinomycin, are known to promote self-assembly by stacking with GQ monomers.^16–19^ However, in long telomeric sequences the flexibility of GQ multimers is critical for determining whether these drugs can be accommodated within the inter-GQ junctions and promote stacking interactions.

To better understand the conformation of GQ multimers, whose structural properties remain largely unresolved,^20^ we employed a Small-Angle X-ray Scattering (SAXS)-guided approach combined with extremely coarse-grained (ECG) Monte Carlo simulations on human telomeric sequences of varying lengths. This allowed us to characterize the large-scale structural properties of telomeric GQ multimers in solution and the destabilizing character of the interaction between their units. On the basis of these findings, we introduced a helix–coil model to describe the stacking–unstacking equilibrium. Our integrated approach, combining SAXS, ECG simulations, and the helix–coil model, can be extended to other biophysical systems, including GQ multimers in biologically relevant RNA regions,^21^ stacked and unstacked domains in single-stranded DNA,^22^ and weakly interacting intrinsically disordered proteins.^23^

Experimental SAXS data on telomeric sequences were retrieved from the online database SASBDB.^24^ Apart from the monomer 2JSL (TAG_3_(T_2_AG_3_)_3_T_2_), the chosen higher-order sequences are Tel48 (T_2_AG_3_)_8_, Tel72 (T_2_AG_3_)_12_ and Tel96 (T_2_AG_3_)_16_, which correspond to 2–4 GQ units, respectively. Monsen et al. reported that these extended telomeric sequences exist in a mixture of approximately 25:75 hybrid-1 and hybrid-2 topologies.^14^ SAXS is a low-resolution technique that is well-suited for investigating the large-scale structure of GQ multimers in solutions at the nanometer scale, despite being unable to capture structural information at the atomic level. Obtaining reliable structural insights from SAXS patterns of GQ multimers requires the intricate integration of ab initio space-filling models with all-atom molecular dynamics (MD) simulations.^14^ We recently showed that SAXS-guided extremely coarse-grained (ECG) simulations can provide a computationally efficient quantitative description of stacking energetics and flexibility of GQ trimers, while neglecting fine details at the level of GQ topologies.^17^ Here, the same approach is applied to describe multimers consisting of a number of GQ units up to *n* = 4. These units are approximated in the ECG model as Hard Cylinders (HCs), held together through an infinite square well interaction patch located on the bases’ edge mimicking TTA linkers. Stacking interactions between adjacent HCs, on the other hand, are implemented by a finite square well potential acting on the bases’ centers.^17^ The depth *u*_0_ of the well is controlled within the simulations via the adimensional effective temperature T* = *k*_*B*_ *T*/*u*_0_.

The best match between the numerical and experimental profiles is obtained by finely exploring the phase space of the simulation parameters, as reported in the Supporting Information. The SAXS data and the best-fitting curves for all the telomeric sequences are shown in Figure 1. The dimensions of the HC unit were found to be the same for all the sequences, with values of R = 1.48 *nm* for the radius and *H* = 2.21 *nm* for the height. The fact that the same HC dimensions are associated with the GQ units of multimers with a length-dependent mixture of hybrid-1 and hybrid-2 topologies^14^ is due to the difficulty in distinguishing different GQ conformers using SAXS, which is a low-resolution structural technique. Regarding the effective temperature governing the interaction at the junction between the HCs, we found the best match to the experimental data to be T* = 0.190 for all the investigated sequences. This high value of T* is compatible with weak inter-GQ attractive interactions. The estimated fractions of bonded sites corresponding to the value of T* obtained from ECG simulations are 31.5 ± 0.1%, 35.0 ± 0.1%, and 36.9 ± 0.1% in the case of Tel48, Tel72, and Tel96, respectively.

**Figure 1:**
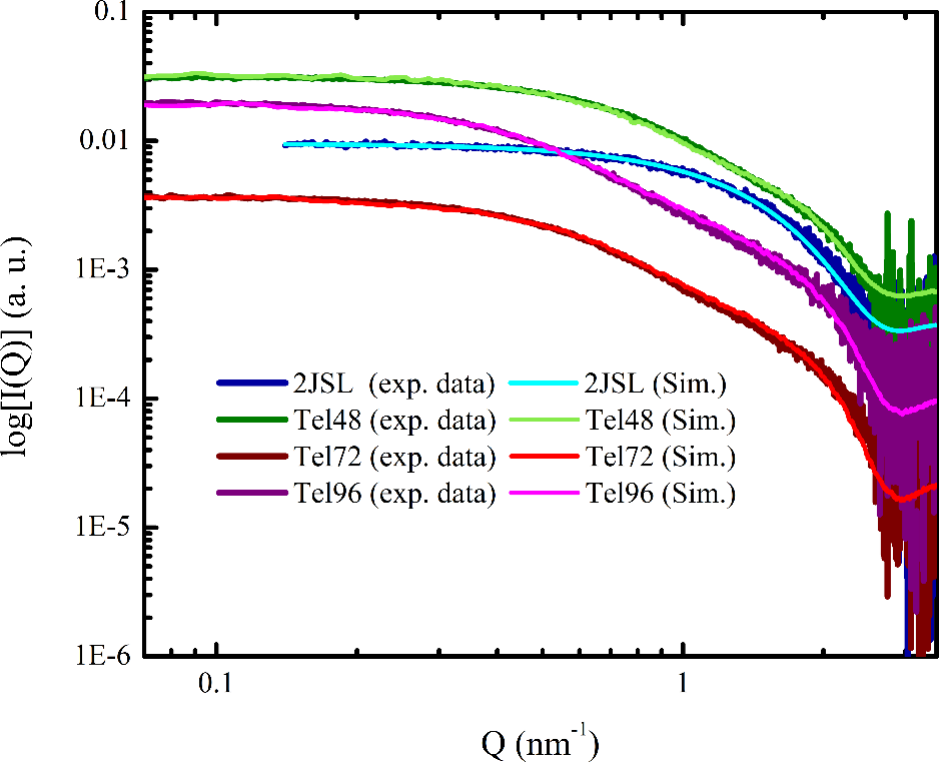
SAXS profiles of 2JSL (blue) Tel48 (green), Tel72 (red), and Tel96 (purple) along with the best matching simulated curves (cyan, light green, light red, and magenta, respectively).

To assess the degree of flexibility at the level of inter-GQ junctions, we computed the distribution of the angle β formed by the central axes of two consecutive HCs. For the best fitting simulation, this distribution (shown in Figure 2, orange line) is a linear combination of the same distributions at very low T* (entirely stacked multimers) with weight *p* = 0.310 and at infinite T*, i.e., in the absence of stacking interactions (entirely beads-on-a-string multimers) with weight 1 – *p*. In the latter case, we estimated that only 0.2% of the inter-GQ junctions adopt a geometry that would be considered bonded in the presence of stacking interactions, which reveals that the system is very unlikely to explore stacked conformations by chance. Therefore, once the size of the HCs and the range of the stacking force are determined, the estimated fractions of bonded sites only depend on the strength of inter-GQ interactions. This approach supports the idea that only a moderate number of dimers explore conformations with closed inter-GQ junctions, as the narrow β distribution peaked at about 15 ° associated with stacked HCs only contributes to about 31% of the total number of configurations explored by the system at T* = 0.190 (Figure 2).

**Figure 2:**
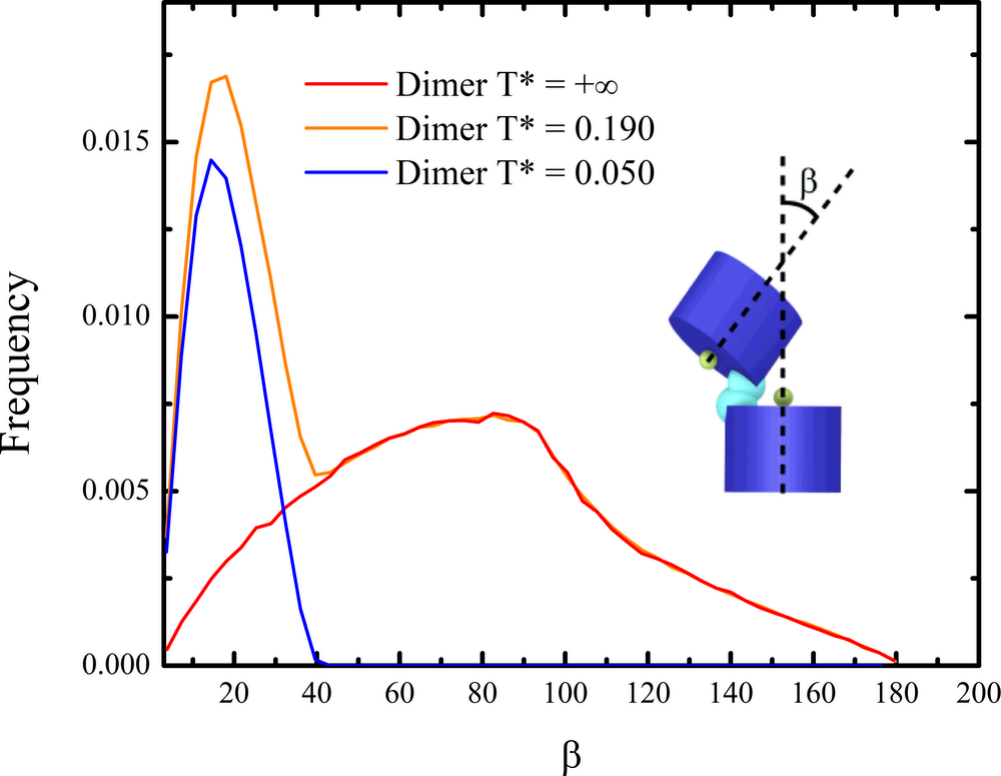
Orange line represents the normalized distribution of the angle β formed between the central axes of two consecutive HCs in dimers at the best fitting effective temperature T* = 0.190. This distribution can be obtained as a linear combination of the same distributions at low T* (blue curve, T* = 0.050) and high T* (red curve, T* = +∞) with weights equal to, respectively, 0.31 and 0.69. The normalized low and high T* curves in the figure are multiplied by the corresponding weight, so that their sum correctly reproduces the orange curve. Only the case of Tel48 is shown, as Tel72 and Tel96 yield much similar results.

Since the human telomeric overhang can form higher-order structures comprising more than 4 GQ units, we developed a model based on the results obtained by combining SAXS and ECG simulations to quantitatively assess the propensity of long multimers to adopt stacked or unstacked conformations. Therefore, we assume that an ensemble of multimers containing *n* GQ units can be described as a system of *n* HC units, each existing in one of two states: stacked/bonded (B) or unstacked/unbonded (U). An HC unit is defined as stacked if its bond with the preceding HC unit is active. Traditionally, the helix–coil transition in polypeptide chains has served as the prototypical example of such biophysical systems. The study of this phenomenon has led to the development of several statistical models, the most well-known likely being the one proposed by Zimm and Bragg.^25^ In this model, the helix–coil transition is characterized by two parameters: *s*, the statistical weight associated with adding a unit in the helical conformation, given that the previous unit is also helical; and σ, which accounts for nucleation by introducing an abnormal reduction in the statistical weight for the first helical unit following a coil segment in the chain.^25^ Inspired by these models, we propose a simple phenomenological description of GQ-multimers. We assume that the first HC unit is unstacked. Upon adding the second unit, we assign a statistical weight of 1 to the state U and a statistical weight of *s* to the state B. As more units are added to the GQ multimer, aggregate statistical weights of the *i*-th unit can be obtained as the product of the aggregate statistical weights of the (*i* – 1)-th unit by the following statistical weights: *q*(*B*|*N*) = *q*(*B*|*B*) = *s* for adding a stacked unit, *q*(*N*|*B*) = 1 for adding an unstacked unit following a stacked unit, and *q*(*N*|*N*) = σ for adding an unstacked unit following another unstacked unit.

The parameter *s*, representing the contribution of a stacked junction to the partition function relative to an unstacked one, is quite similar to the parameter used by Zimm and Bragg. On the other hand, σ serves as a corrective term or entropic penalty, reducing the number of possible configurations for two consecutive unstacked junctions due to potential overlaps between second-nearest neighbors. This physical interpretation is quite different from the one associated with the corresponding parameter in the Zimm–Bragg model.

By introducing the transfer matrix:

1 $$G=\left(\begin{array}{cc} q\left(N|N\right) & q\left(N|B\right)\\ q\left(B|N\right) & q\left(B|B\right) \end{array}\right)=\left(\begin{array}{cc} \sigma & 1\\ s & s \end{array}\right)$$

the aggregate statistical weights for the *n*-th GQ unit of the multimer, which can be expressed as the components of the vector ***a***_*n*_, are given by

2 $$a_{n}={\mathrm{Ga}}_{n-1}=G^{n-2}a_{2}$$

where ***a***_2_ = (1, *s*)^*T*^ contains the statistical weights of the second unit.

To obtain the partition function *Q*_*n*_ for a multimer of *n* GQs, it is sufficient to sum all the components of the vector ***a***_*n*_, which can be done by multiplying on the left by the vector **ω** = (1, 1)^*T*^. By diagonalizing the transfer matrix ***G*** we obtain

3 $$Q_{n}=\omega^{T}T\Lambda^{n-2}T^{-1}a_{2}$$

where the columns of ***T*** are the eigenvetors of ***G*** and the diagonal matrix **Λ** contains the eigenvalues of ***G***, which are equal to

4 $$\lambda_{0,1}=\frac{1}{2}\left\{s+\sigma \pm \sqrt{{\left(s-\sigma \right)}^{2}+4s}\right\}$$

By using these values for λ_0,1_ and the corresponding eigenvectors for ***T***, eq 3 becomes (λ_0_ > λ_1_):

5 $$Q_{n}\left(s,\sigma \right)=\frac{1}{\lambda_{1}-\lambda_{0}}\left\{\left(\lambda_{1}^{n}-\lambda_{0}^{n}\right)+\left(\lambda_{1}^{n-1}-\lambda_{0}^{n-1}\right)\left(1-\sigma \right)\right\}$$

To determine the values of the *s* and σ parameters that fully define *Q*_*n*_, we employed the following strategy. We estimated the average fraction of stacked GQs *f*_*n*_ as a function of *n* from the conformations obtained by the ECG simulations used to reproduce the SAXS experimental data. Then, we exploited the relationship:^26^

6 $$f_{n}=\frac{s}{n-1}\frac{\partial }{\partial s}\ln Q_{n}$$

to fit *f*_*n*_ as a function of *Q*_*n*_ as defined by eq 5 and determined the parameters *s* = 0.455 ± 0.008 and σ = 0.76 ± 0.02. Once *Q*_*n*_ is determined, all the relevant thermodynamic quantities of the system can be calculated. As an example, we obtained the specific heat as a function of the temperature and predicted the presence of a transition from a fully stacked to partially beads-on-a-string system at about 250 K (see Supporting Information). Furthermore, assuming that the same values of s and σ remain valid for longer sequences—which is reasonable given the weak inter-GQ stacking—and that the overlap between cylinders separated by more than two units can be neglected, we can directly estimate *f*_*n*_ for arbitrarily long sequences (see Figure 3, panel (a)). Knowing *f*_*n*_ can be extremely useful for pharmaceutical purposes as it represents the population of targets for ligands recognizing specifically the cleft between two stacked GQs.^27^ It has been recently reported that, consistent with the length of the TTA linker, the size of such a cleft ranges from 7 Å to 10 Å and that it is highly electronegative, thus having the potential to accommodate GQ-interacting ligands.^14^ On the other hand, to estimate the number of binding sites for drugs that require a large pocket between adjacent GQs, such as possibly in the case of bifunctional ligands,^28^ one can easily calculate the fraction of unstacked GQ pairs as 1 – *f*_*n*_. Analogously, *Q*_*n*_ can be used to compute the fractions of stacked or unstacked multiplets of GQs. As an example, in Figure 3, panel (b) we report the fraction of *m* unstacked GQs *f*_*u*,*n*_^(*m*)^ in a chain with *n* units, which can optimally interact with drugs targeting *m* = 3, 4, 5 consecutive inter-GQ large pockets.^27^ The trend of *f*_*n*_^(*m*)^ suggests that there is a significant fraction of such potential targets even for values of *m* as high as 5. We remark that the effect of inter-GQ overlaps, which is considered in the model through the parameter σ, is the reduction of the fraction of unstacked sites in a multimer. As the number of GQ units increases, the number of configurations that are not allowed due to overlaps between GQ units increases, while the number of unstacked inter-GQ junctions decreases.

**Figure 3:**
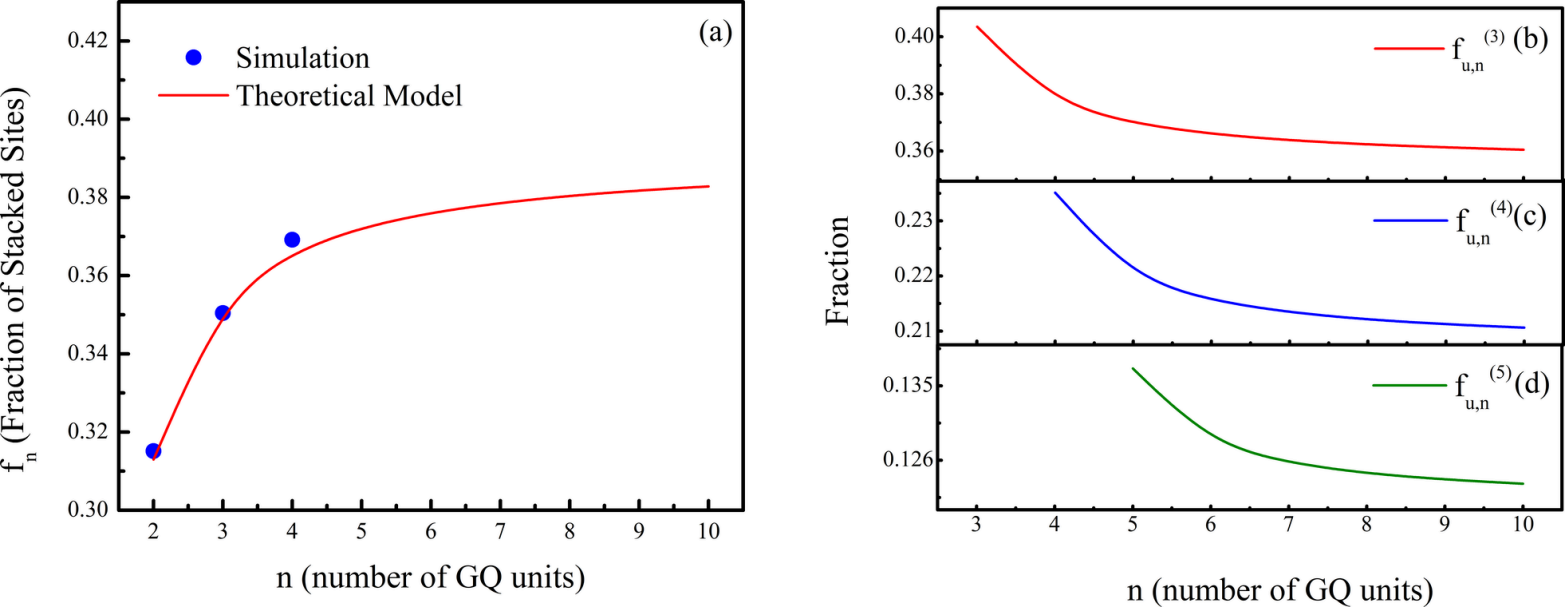
Panel (a): fraction of stacked inter-GQ junctions *f*_*n*_ as a function of the multimer’s number of GQ units *n* (blue circles) along with the best fit using eq 6 (red solid line). The values of *f*_*n*_ as predicted by the model are shown up to *n* = 10. Panels (b), (c), and (d): fraction of *m* = 3, 4, 5 consecutive unstacked GQ units *f*_*u*,*n*_^(*m*)^ as a function of *n* (red, blue, and green solid lines, respectively).

An ensemble of GQ *n*-mers can be described as a mixture of stacked and unstacked sites at equilibrium. The fraction *f*_*n*_ can then be used to determine the free energy *ΔG*_*s*_ for the formation of a stacked site using the following equation.

7 $$\Delta G_{s}\left(n\right)=-k_{B}T\left(n-1\right)\log\left(\frac{f_{n}}{1-f_{n}}\right)$$

Within this context, the quantity exp­(−*ΔG*_*s*_(*n*)/*k*_*B*_T) can be interpreted as the kinetic rate constant for the conversion of unstacked sites into stacked ones. From eq 7 it is also possible to show that *s* = exp­(−*ΔG*_*s*_(2)/*k*_*B*_ *T*). This arises from the fact that the dimer’s partition function *Q*_2_ depends only on the parameter *s*, while in longer multimers the depndence of *Q*_*n*_ on σ introduces an additional entropic contribution (see Supporting Information). As reported in Table 1, the values of *ΔG*_*s*_ obtained using eq 7 are positive, thus suggesting a prevalent destabilizing role of the entropic contribution in the stacking interaction. This stems from the fact that, despite the presence of an attractive force within the model, the system is much more prone to explore unstacked configurations at equilibrium. Furthermore, although these values increase as a function of the number of GQ units, the contribution of the single inter-GQ junction *ΔG*_*s*_/(*n* – 1) decreases with *n*. In fact, each additional GQ unit in the *n*-mer contributes a progressively smaller destabilizing contribution to *ΔG*_*s*_, consistent with the increase in the number of stacked sites as *n* grows. This is due to an excluded-volume effect where the number of available unstacked configurations of nonconsecutive HC units is reduced in longer multimers. In the past, calorimetry and spectroscopy melting experiments^12,29^ have been used to estimate the free energy of coupling *ΔG*_*coupling*_ between GQ units in multimers, through the relationship:

8 $$\Delta G_{coupling}\left(n\right)=\Delta G_{folding}\left(n\right)-n\Delta G_{folding}\left(n=1\right)$$

where *ΔG*_*folding*_(*n*) and *ΔG*_*folding*_(*n* = 1) are, respectively, the folding free energies of the GQ *n*-mer and of the GQ monomer.

**Table 1:** Values of *ΔG*_*s*_ Obtained Using eq 7 Are Presented as a Function of the Number of GQ Units, *n* ^a^

*n*	*ΔG*_*s*_	*ΔG*_*s*_/(*n*–1)	*ΔG*_*coupling*_
2	+0.452 ± 0.004	+0.452 ± 0.004	+1.64
3	+0.719 ± 0.007	+0.360 ± 0.003	+2.28
4	+0.94 ± 0.01	+0.312 ± 0.003	+3.24

a Additionally, the values of *ΔG*_*s*_ per interaction site are provided, along with the values of *ΔG*_coupling_ from Yu et al.^12^ All values are expressed in kcal·mol^–1^.

The *ΔG*_*s*_(*n*) values derived from our model are quite smaller than *ΔG*_*coupling*_(*n*),^12^ even though both quantities are positive in sign, which is consistent with a destabilizing effect. One could be tempted to directly compare *ΔG*_*coupling*_(*n*) with *ΔG*_*s*_ (*n*). However, *ΔG*_*s*_ is just the free energy for the conversion of unstacked sites into stacked ones, while *ΔG*_*coupling*_ contains the free energy of the ensemble of GQ *n*-mers. Furthermore, the folding free energy also depends on the topology of the GQ units,^29^ which, as we mentioned above, in multimers can be different from that of the monomers.^14^ In addition, the contributions from specific interactions between the solvent and folded/unfolded multimers or monomers should also be taken into account. In this regard, it has been proposed that dehydration of the interior of the GQ units, accompanied by a water molecules uptake at the level of the TTA linkers, plays a key role in the destabilization of the GQ multimer.^12^ As a consequence, the interplay of all these factors is quite complex and elaborating a model that yields stacking free energies that are directly comparable with experimentally derived quantities is not a trivial task. Nonetheless, the proposed Zimm–Bragg-like model offers valuable insights into the energetics of GQ multimers, as evidenced by the observed trends in *ΔG*_*s*_ and *ΔG*_*s*_/(*n* – 1). The destabilizing free energy of the stacking between GQs identified in the present study could play significant and multifaceted biological roles. The transition between folded and unfolded states of GQs which may be more rapid in partly unstacked than in fully stacked ensembles, could provide telomeres with an optimal structural flexibility to facilitate the interaction with the telomere-binding proteins that are key for maintaining telomere integrity, such as shelterin components (e.g., TRF1, TRF2, POT1). Additionally, destabilizing stacked GQ multimers may serve as a regulatory switch for telomerase activity. While folded GQs inhibit telomerase binding, partially unstacked and unfolded ensembles might expose regions of telomeric DNA, making them accessible to telomerase.

As stated before, several experimental studies support the view where telomeric GQ multimers are arranged in a noninteracting, beads-on-a-string configuration,^11,12,30,31^ even though this point is still a matter of debate.^20^ The destabilizing contribution of the stacking free energy that we derived implies that GQ multimers in single-strand telomeric regions are composed prevalently of unstacked units. Despite the quite weak inter-GQ attractive interactions, thermal fluctuations are not sufficient to drive significant bending of short GQ multimers. Indeed, we found that only a small fraction — less than 2% — of the GQ multimeric configurations sampled in the ECG simulations spontaneously bend by approximately 180 ° along the arc of a semicircle with a given radius (see Supporting Information). This result supports the notion that inducing a U-turn bend in short GQ multimers—necessary to connect the terminal 3′ d­(TTAG) repeat (bound by POT1) with the shelterin complex—requires an external energy input, unless the telomeric sequence exists in a single-stranded state.^14^ Overall, our findings have important implications not only for the understanding of telomere biology and the design of therapeutic ligands targeting higher-order telomeric GQs, but also beyond. As an exemple, the proposed integrated approach could be extended to higher-order GQ structures formed in gene promoters, provided that ECG simulations are adapted to account for the presence of the complementary DNA strand. A prototypical case is that of the G-rich domain of the KIT promoter, where the simultaneous formation of three GQs has been shown by using a magnetic tweezer approach at the single-molecule level.^32^ Furthermore, the present method could be employed in other biophysically relevant systems characterized by interacting units in multimeric superstructures, such as intrinsically disordered proteins.^33^

## Supplementary Material



## Notes

Monte Carlo Simulations and Fitting Procedure, Specific Heat of Inter-GQ Interactions, Alternative Derivation of the Partition Function, Bending of the GQ-Multimers (PDF)
