# Supplementary material for: Destabilizing Interactions in Human Telomeric G‑Quadruplex Multimers
Source: J Phys Chem Lett. 2025 May 23;16(22):5435–40. doi: 10.1021/acs.jpclett.5c01100 (PMC12147199; doi:10.1021/acs.jpclett.5c01100)
Supplement: Supplementary file 1 [file jz5c01100_si_001.pdf]

Supporting Informtaion: Destabilizing Interactions in Human Telomeric G-Quadruplex Multimers

Luca Bertini,[†] Mattia Trapella,[‡] Deniz Mostarac,[¶] Valeria Libera,[‡] Caterina Petrillo,[‡] Cristiano De Michele,[¶] Lucia Comez,[*,§] and Alessandro Paciaroni[*,‡]

†*Department of Physics and Geology, University of Perugia, via Alessandro Pascoli, 06123 Perugia, Italy*

‡*Department of Physics and Geology, University of Perugia, via Alessandro Pascoli, 06123 Perugia, Italy*

¶*Department of Physics, University of Rome La Sapienza, 00185 Rome, Italy*

§*CNR-IOM c/o Department of Physics and Geology, University of Perugia, 06123 Perugia, Italy*

E-mail: comez@iom.cnr.it; alessandro.paciaroni@unipg.it

Monte Carlo Simulations and Fitting Procedure

The ECG model employed in the present work is based on a simplified representation of the GQ multimers where each GQ unit is represented as a Hard Cylinder, as described in the main text and in our previous work.[1] The width of the square well potentials implementing the TTA linker and the stacking interactions were fixed to 0.53 nm and 0.265 nm, respectively. In order to reduce the number of parameters, the aspect ratio K was introduced such that the radius and height of the HCs were, respectively, $R = R_0K$ and $H = H_0/K$ with $R_0 = 1.06$

nm and $H_0 = 3.1$ nm. The Monte Carlo (MC) simulations were performed in the NVT* ensemble, with 6000 HCs (corresponding to 3000 dimers, 2000 trimers and 1500 tetramers) in a 500x500x500 nm^3 box. The system was thermalized for at least 10^6 MC steps. A recently developed algorithm checking the overlap between HCs was employed to reject MC moves leading to overlapping units.[2,3] The simulated scattering intensity was computed from the obtained HC configurations by replacing each HC with a set of 100 scattering points randomly placed with uniform number density.[4] Subsequently, a grid of simulated SAXS curves was produced in the parameters K (ranging from 0.80 to 1.80, with steps $\Delta K = 0.05$) and T* (ranging from 0.08 to 0.20, with steps $\Delta T^* = 0.005$). Finally, we selected the best fitting simulated intensity by minimizing the reduced chi-square. We found that all the investigated systems were best described by the combination T$^* = 0.190$ and K = 1.40, corresponding to the HC dimensions $R = 1.48$ nm and $H = 2.21$ nm.

Specific Heat of inter-GQ Interactions

The main advantage of a model providing an analytical expression for the partition function is that it allows to retrieve relevant thermodynamic functions. In particular, the contribution of the stacking interactions to the specific heat at constant volume can be computed as:

$$C_V^{ST}(T) = k_B \left(2T \frac{\partial}{\partial T} \ln(Q_n) + T^2 \frac{\partial^2}{\partial T^2} \ln(Q_n) \right), \tag{S1}$$

where Q_n is the partition function from our Ising-like model (Eq. 5 of the main text). In Figure S1 we report C_V as a function of temperature. Interestingly, the values of C_V are significantly lower than the C_P values for the melting of GQ multimers obtained by *Petraccone et al.* using DSC.[5] This result is again consistent with weak inter GQ stacking interactions.

Furthermore, our models predicts the presence of a transition from an entirely stacked ensemble of GQ-multimers at lower temperatures to a semiflexible, partially beads-on-a-

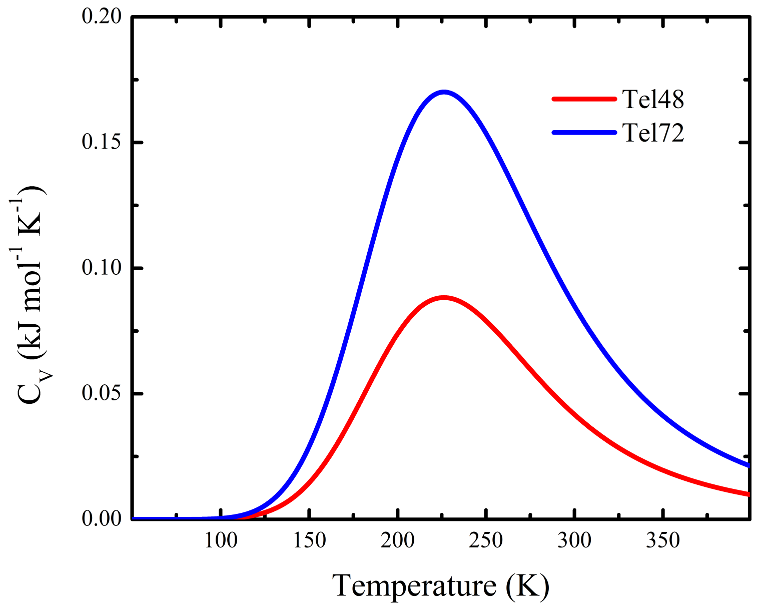

Figure S1: Contribution of the stacking interactions to the specific heat C_V as a function of temperature for Tel48 (red) and Tel72 (blue). The peak at $T \simeq 230$ K marks the predicted transition from entirely stacked to partially beads-on-a-string ensembles.

string one at higher temperatures, as indicated by the peak in $C_V(T)$ around $T \simeq 230$ K. As the presence of this transition has not yet been experimentally confirmed, our findings highlight the need for further investigation.

Alternative Derivation of the Partition Function

From our model it is possible to estimate the free energy of an ensemble of GQ n-mers at equilibrium. In the case of dimers, this can be done by writing the partition function as:

$$Z_2 = \int_B d\vec{q}_B e^{-E_B/k_BT} + \int_N d\vec{q}_N e^{-E_N/k_BT}, \tag{S2}$$

where the first integral is computed over the degrees of freedom corresponding to a stacked dimer ($\vec{q}_B$), while the second integral is over the degrees of freedom corresponding to unstacked configurations ($\vec{q}_N$). As the energy of a stacked unit is $E_B = -u_0$ and that of an

unstacked unit is $E_N = 0$, we may write:

$$Z_2 = e^{u_0/k_BT} \int_B d\vec{q}_B + \int_N d\vec{q}_N. \tag{S3}$$

The two integrals now correspond to the volumes in the phase space of stacked (V_B) and unstacked (V_N) configurations, respectively. Now, by dividing the partition function by V_N we get an equivalent partition function:

$$Q_2 = \frac{V_B}{V_N} e^{u_0/k_BT} + 1. \tag{S4}$$

By defining the entropy difference between stacked and unstacked configurations as $\Delta S_s(2) = k_B \ln(V_B/V_N)$, and taking into account the fact that $\Delta E(2) = E_B - E_N = -u_0$, we obtain:

$$Q_2 = e^{-\Delta G_s(2)/k_BT} + 1 = s + 1, \tag{S5}$$

which is equivalent to the one obtained using Eq. 5 of the main text. By applying Eq. 6 of the main text, the fraction of stacked sites within an ensemble of GQ dimers is found to be $f_2 = s/(1+s)$. It is worth of note that by inserting this result into Eq. 7 of the main text we obtain again $s = \exp(-\Delta G_s(2)/k_BT)$.

In the case of trimers, a similar approach can be adopted. Here we make the same approximation described in the main text, i.e. the effect of the addition of a GQ unit depends on the state of the previous inter-GQ junction only in the case of two contiguous unstacked junctions. In this case, the probability $q(N|N)$ is lower than $q(N|B)$ by a factor $\sigma < 1$. This stems from the fact that two contiguous interaction sites can lead to the overlap of the i-th GQ unit with the $(i-2)$-th one, so that the volume in the phase space of the NN configuration can be written as $V_{NN} = \sigma V_N V_N$. In the other configurations (BB, NB and BN), on the other hand, the state of the j-th junction is independent of the state of the previous one, so that we may assume that $V_{BB} = V_B V_B$ and $V_{NB} = V_{BN} = V_B V_N$. Therefore,

the partition function of the trimer can be written as:

$$Z_3 = V_B^2 e^{2u_0/k_BT} + 2V_B V_N e^{u_0/k_BT} + \sigma V_N^2. \tag{S6}$$

This time, we can get an equivalent partition function by dividing by V_N^2:

$$Q_3 = (V_B/V_N)^2 e^{2u_0/k_BT} + 2(V_B/V_N)e^{u_0/k_BT} + \sigma, \tag{S7}$$

and since $s = \exp(-\Delta G_s(2)/k_BT)$ we now obtain:

$$Q_3 = s^2 + 2s + \sigma, \tag{S8}$$

which is again equivalent to the one derived from Eq. 5 of the main text.

Bending of the GQ-Multimers

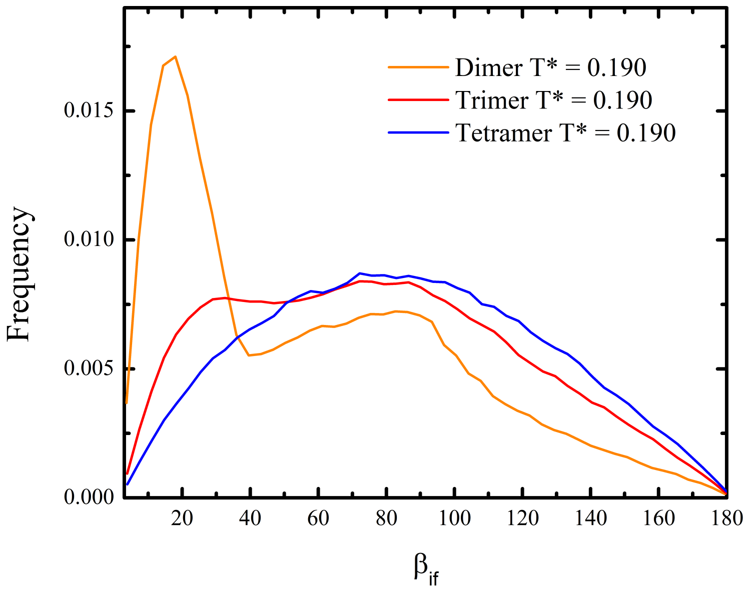

Figure S2: Distribution of the angle β_{if} formed between the central axes of the first and last HC in dimers (orange curve), trimers (red curve) and tetramers (blue curve).

In the main text, we show that the distribution of the angles β formed by the central axes

of two consecutive HCs in a multimer can offer insight into the flexibility of the structures under investigation. In Fig. S2 we also show the distribution of the angle β_{if} formed by the central axes of the first and last HC in dimers, trimers and tetramers. As the number of GQ multimers increases the features of stacking interactions and inter-GQ overlaps, which are visible in the case of dimers, gradually disappear from the distribution. In any case, the number of configurations corresponding to values of β_{if} close to 180° is quite low. To make a shallow estimate, we computed the area under the curves in the range [165°:180°], which corresponds to the fraction of configurations with β_{if} falling into that range. We found that this fractions are 0.8%, 1.3% and 1.8% in the case of dimers, trimers, and tetramers, respectively. These configurations, or at least part of them, correspond to GQ multimers bending into a semi-circle, a conformation that has been estimated to be too energetically demanding to occur spontaneously at room temperature.[6] Therefore, our estimates support the idea that excessive bending of GQ multimers is a process that requires an external energy input to occur.

References

(1) Rosi, B. P.; Libera, V.; Bertini, L.; Orecchini, A.; Corezzi, S.; Schirò, G.; Pernot, P.; Biehl, R.; Petrillo, C.; Comez, L.; others Stacking Interactions and Flexibility of Human Telomeric Multimers. *Journal of the American Chemical Society* **2023**, *145*, 16166–16175.

(2) Orellana, A. G.; Romani, E.; De Michele, C. Speeding up Monte Carlo simulation of patchy hard cylinders. *The European Physical Journal E* **2018**, *41*, 1–10.

(3) Orellana, A. G.; Michele, C. D. Algorithm 1010: Boosting efficiency in solving quartic equations with no compromise in accuracy. *ACM Transactions on Mathematical Software (TOMS)* **2020**, *46*, 1–28.

(4) Pal, A.; De Filippo, C. A.; Ito, T.; Kamal, M. A.; Petukhov, A. V.; De Michele, C.; Schurtenberger, P. Shape matters in magnetic-field-assisted assembly of prolate colloids. *ACS nano* **2022**, *16*, 2558–2568.

(5) Petraccone, L.; Spink, C.; Trent, J. O.; Garbett, N. C.; Mekmaysy, C. S.; Giancola, C.; Chaires, J. B. Structure and stability of higher-order human telomeric quadruplexes. *Journal of the American Chemical Society* **2011**, *133*, 20951–20961.

(6) Monsen, R. C.; Chakravarthy, S.; Dean, W. L.; Chaires, J. B.; Trent, J. O. The solution structures of higher-order human telomere G-quadruplex multimers. *Nucleic Acids Research* **2021**, *49*, 1749–1768.
